# Successful Urethrorectal Fistula Repair Using GelPOINT Path Anal Access Sheath in Pelvic Fracture Urethral Injury

**DOI:** 10.1002/iju5.70028

**Published:** 2025-04-27

**Authors:** Wataru Tanaka, Akihiro Kanematsu, Naohito Beppu, Kenichiro Kawai, Shinpei Yoshioka, Kimihiro Shimatani, Toeki Yanagi, Masao Kakibuchi, Masataka Ikeda, Shingo Yamamoto

**Affiliations:** ^1^ Department of Urology Hyogo Medical University Hyogo Japan; ^2^ Division of Colorectal Surgery, Department of Gastrointenstinal Surgery Hyogo Medical University Hyogo Japan; ^3^ Plastic Surgery Hyogo Medical University Hyogo Japan

**Keywords:** rectourethral fistula, transanal access sheath, urethroplasty

## Abstract

**Introduction:**

We present a case of rectourethral fistula complicating urethral trauma, successfully treated using a transanal exposure device.

**Case Presentation:**

A male in his twenties was referred to us with a pelvic fracture urethral injury. Preoperative imaging revealed an urethrorectal fistula. Excision and primary anastomosis urethroplasty was performed transperineally. The fistula was primarily closed transanally via GelPOINT Path access sheath, which provided wide access to the anal canal. The closure was reinforced perineally, with a gracilis muscle flap interposition. Postoperative urethral patency was excellent without recurrent fistula.

**Conclusion:**

This method could be an effective alternative for urethrorectal fistula repair.

**Summary**

- The transanal approach with a GelPOINT path access sheath was useful for treating a rectourethral fistula complicating urethral injury.

**Abbreviations**
EPA: excision and primary anastomosis
PFUI: pelvic fracture urethral injury
POD: post‐operative day
URF: urethrorectal fistula

## 1 Introduction

Pelvic fracture urethral injury (PFUI) is associated with 1.4%–2% of pelvic fractures and affects the membranous to bulbar urethra. The standard procedure is excision and primary anastomosis (EPA) urethroplasty, which involves extensive scar resection followed by end‐to‐end anastomosis. Urethrorectal fistula (URF) is one of the conditions that complicate EPA for PFUI [1]. Here, we present such a case, in which an anal access sheath was employed to attain better surgical exposure of URF.

## 2 Case Presentation

**FIGURE 1:**
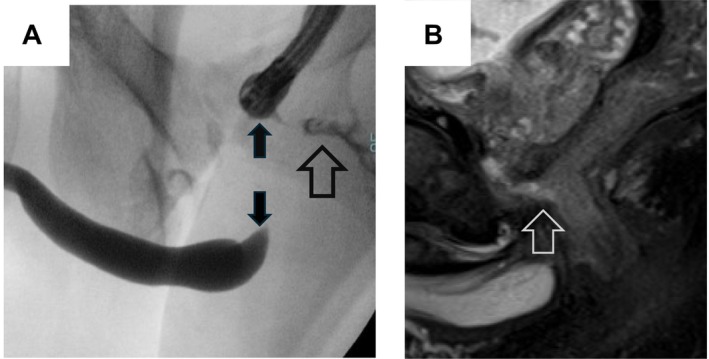
(A) Combined urethrogram shows urethral gap and urethrorectal fistula. (B) T2‐weighed magnetic resonance imaging showing urethrorectal fistula. Open arrow: Urethrorectal fistula; Closed arrow: Urethral gap.

A male in his twenties was crushed by heavy machinery during construction work, resulting in an open pelvic fracture, urethral injury, and rectal injury. The patient was sent to a local emergency center, where he underwent sigmoid colostomy and suprapubic cystostomy tube placement. Seven months after the initial injury, the patient was referred to our hospital by local urologists for urethroplasty after closure of the sigmoid colostomy. At his initial visit, the patient reported “leakage of urine from the anus.” Combined cystourethrography at our hospital showed a rectourethral fistula at membranous urethra and urethral gap approximately 3 cm in width (Figure 1A). Pelvic magnetic resonance imaging revealed similar findings (Figure 1B). Upon the diagnosis of PFUI with concomitant URF, we planned to perform EPA with simultaneous fistula closure.

**FIGURE 2:**
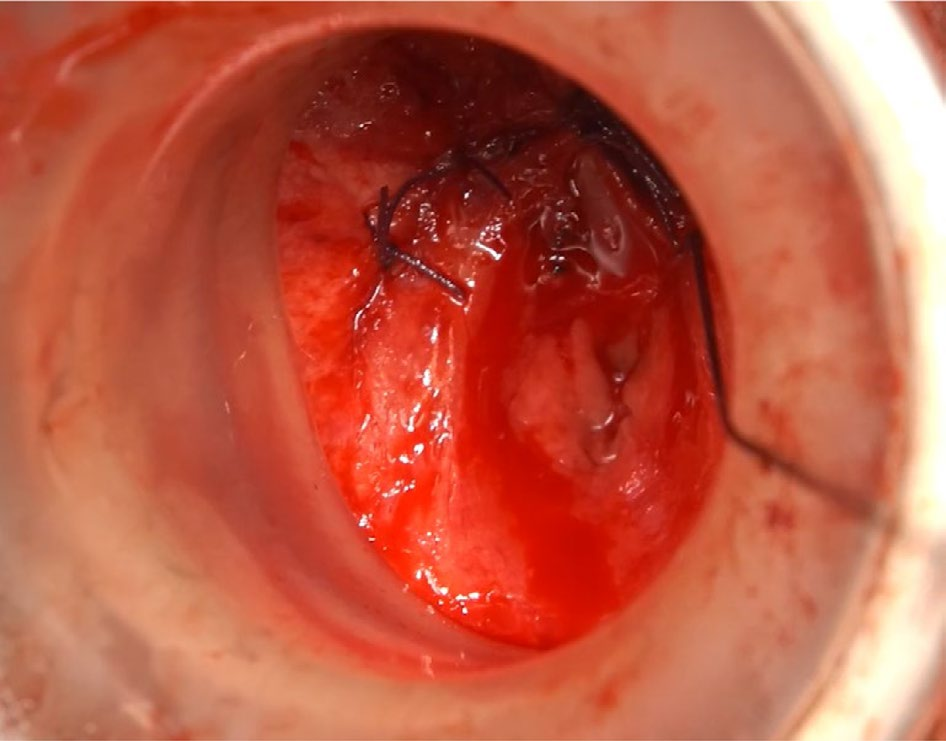
After completion of transrectal closure of urethrorectal fistula via GelPOINT. Closure sutures are visible under direct vision.

**FIGURE 3:**
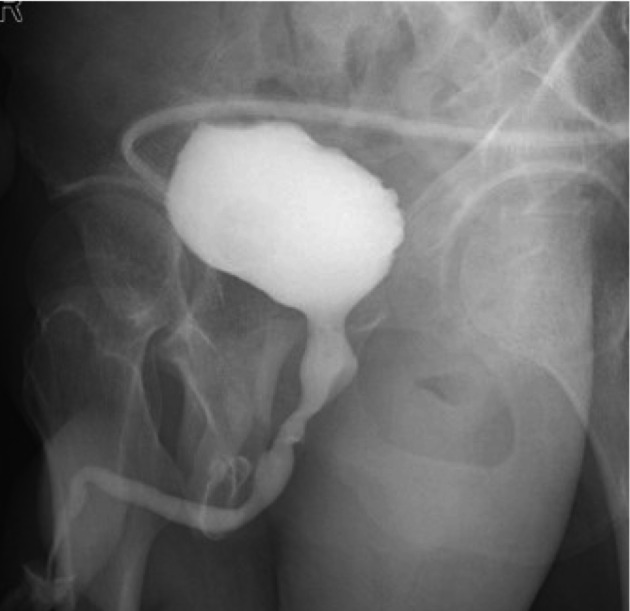
Postoperative voiding cystourethrogram showing patent anastomosis with no residual urethrorectal fistula on postoperative Day 25.

Surgical procedure: With the patient in the lithotomy position, a perineal midline incision was made to access the urethra. Dense scar due to trauma was removed until exposure of the tip of the flexible cystoscope inserted from the cystostomy tract. During this procedure, the fistulous tract was encountered and removed. The colorectal surgical team deployed a GelPOINT Path access sheath (Applied Medical Japan, Tokyo, Japan) to expose the anal canal. The unhealthy tissue around the fistula was removed from the perineal side and URF was closed from the rectal side using intermittent 3‐0 polyglactin sutures (Figure 2). The urology team placed additional sutures from the perineal side to reinforce the closure, followed by an end‐to‐end urethral anastomosis. Furthermore, the plastic surgery team harvested a gracilis muscle flap from the patient's right thigh, which was transposed into the perineal wound and sutured to the Denonvilliers' Fascia, forming a barrier layer between the closed URF and urethral anastomosis. Finally, the colorectal surgeons created an ileostomy for fecal diversion. The total surgery duration was 8 h and 40 min, with a blood loss of 290 mL. The indwelling urethral catheter was removed on POD25. Voiding cystourethrography revealed a wide patent anastomosis without leakage or recurrent URF (Figure 3). The ileostomy was closed on POD51. No fecal incontinence occurred after surgery (Wexner score: 0). Postoperative uroflowmetry showed a maximal flow rate of 29.7 mL/s, suggesting anatomical success, and the patient continued voiding at a similar level for 1 year.

## 3 Discussion

URF is often caused by prostatectomy or local ablation therapy for the prostate and is rarely traumatic [2]. Common approaches for URF closure are transperineal, transanal (Parks), and transsphincteric (York–Mason) [2]. The transperineal approach is most commonly used, as it allows access to the urethra and facilitates the placement of a barrier layer; however, secure URF closure from the perineal wound is not always warranted [3]. In contrast, the transanal and transsphincteric approaches (York–Mason procedure) can close the URF from the rectal side. Of these two approaches, the York–Mason procedure provides wider exposure of the fistula but requires the patient to be placed in the supine position, making urethral manipulation difficult.

The transanal approach in the lithotomy position is less invasive but is believed to provide poorer exposure than the York–Mason procedure and has not been the mainstay for URF repair. However, colorectal surgeons have recently introduced the GelPOINT Path access sheath for transanal endoscopic surgery to treat early rectal cancer [4] and rectourethral fistula [5], referring to this approach as transanal minimal invasive surgery (TAMIS). Moreover, a study reported that the same sheath allows wide exposure of the rectal wall, not only for endoscopic access but also for open surgical repair of rectal trauma [6]. In our case, we followed the method of that report and combined the transperineal approach for standard EPA with transanal open surgery using the GelPOINT Path sheath. This combination allowed us to close the URF from both sides of the anterior rectal wall and place a gracilis muscle flap as a barrier layer, resulting in a successful simultaneous repair of PFUI and URF. The only drawback of this method is the extra cost of the sheath, but this seems outweighed by the benefit of the excellent exposure.

To the best of our knowledge, this is the first report on the use of this device in the open repair of a URF. Although this method has not yet been applied to URF resulting from different etiologies, such as prostatectomy or pelvic irradiation, its advantages may theoretically be applicable to these cases because many high‐volume centers perform URF repair using the transperineal approach. Therefore, adjunctive repair from the rectal side in the lithotomy position may be a valuable tool for urethral reconstructive surgeons.

In summary, the transanal approach using an anal access sheath provided wide exposure of the URF from the rectal side in the lithotomy position. The present methodology may be an optimal approach for treating URF concomitant with PFUI and should be replicated in more cases and possibly for URF derived from different etiologies.

## Consent

The authors have nothing to report.

## Conflicts of Interest

The authors declare no conflicts of interest.
